# Annotated genome sequences of the carnivorous plant *Roridula gorgonias* and a non-carnivorous relative, *Clethra arborea*

**DOI:** 10.1186/s13104-020-05254-4

**Published:** 2020-09-10

**Authors:** Stefanie Hartmann, Michaela Preick, Silke Abelt, André Scheffel, Michael Hofreiter

**Affiliations:** 1 Institute for Biochemistry and Biology, University of Potsdam, Karl-Liebknecht-Str. 24-25, 14476 Potsdam, Germany; 2 Max-Planck-Institute of Molecular Plant Physiology, Am Mühlenberg 1, 14476 Potsdam, Germany

**Keywords:** Carnivorous plant, *Roridula gorgonias*, *Clethra arborea*, Genome assembly, Transcriptome assembly, Phylogenomics, Orthologous Matrix (OMA) Project

## Abstract

**Objective:**

Plant carnivory is distributed across the tree of life and has evolved at least six times independently, but sequenced and annotated nuclear genomes of carnivorous plants are currently lacking. We have sequenced and structurally annotated the nuclear genome of the carnivorous *Roridula gorgonias* and that of a non-carnivorous relative, Madeira’s lily-of-the-valley-tree, *Clethra arborea*, both within the Ericales. This data adds an important resource to study the evolutionary genetics of plant carnivory across angiosperm lineages and also for functional and systematic aspects of plants within the Ericales.

**Results:**

Our assemblies have total lengths of 284 Mbp (*R. gorgonias*) and 511 Mbp (*C. arborea*) and show high BUSCO scores of 84.2% and 89.5%, respectively. We used their predicted genes together with publicly available data from other Ericales’ genomes and transcriptomes to assemble a phylogenomic data set for the inference of a species tree. However, groups of orthologs showed a marked absence of species represented by a transcriptome. We discuss possible reasons and caution against combining predicted genes from genome- and transriptome-based assemblies.

## Introduction

Although plants can convert water, $\hbox {CO}_2$, and light energy into organic compounds by photosynthesis, they require additional minerals and nutrients for growth and reproduction. Most plants take up these essential compounds from the soil. Several plants in multiple and diverse angiosperm lineages, however, have independently adopted a carnivorous life style [1]: they attract and capture insect prey and absorb essential nutrients from the dead animals. Not surprisingly, plant carnivory has evolved mostly in areas that are low in nutrients, so the increased nutrient availability through predation provides a clear selective advantage.

To study the evolution and molecular adaptations involving plant carnivory, annotated genome data is an essential resource. However, although more than 600 carnivorous plant species have been described [1], sequenced and annotated nuclear genomes of only four of these remarkable plants are currently available [2–5]. For a few additional carnivorous plants, unannotated genome [6] or transcriptome assemblies [7–10] are available. Sequence data for molecular and evolutionary studies in carnivorous plants is therefore clearly lacking. This study contributes the nuclear genomes of two plants within the Ericales: of the carnivorous plant *Roridula gorgonias*, considered by some authors as proto-carnivorous [11], as well as that of a non-carnivorous relative, Madeira’s lily-of-the-valley-tree, *Clethra arborea*. We have used the predicted genes of their genomes for a phylogenomic analysis of plants within the Ericales and conclude that genome-based protein sets, as opposed to incomplete and fragmented transcriptome-based data, are needed for future phylogenomic studies that focus on systematic and functional aspects of this plant group.

## Main text

### Materials and methods

#### Sample collection

*Clethra arborea* Aiton leaves (IPEN number PT-0-B-3250100; source: Madeira, Funchal) were sampled at the Botanical Garden in Berlin on Oct 22, 2018 for estimation of genome size and on Apr 1, 2019 for DNA extraction and sequencing. *Roridula gorgonias* Planch. (IPEN number XX-0-B-0981111; source: Botanical Garden Liberec) was sampled at the Botanical Garden in Berlin on Apr 1, 2019 for DNA extraction and sequencing; leaves were sticky but free of macroscopic insect remains. *C. arborea* leaves for estimation of genome size were kept wrapped in moist paper towels at 4 degrees Celsius until use the next day; young leaves for DNA extraction from both species were separately collected into and stored in liquid Nitrogen until use.

#### Estimation of *C. arborea* genome size

The genome size of *R. gorgonias* was reported to be 186 Mbp [12]. For *C. arborea*, no information about genome size was available, although the plant is known to be diploid with two sets of eight chromosomes [13]. Prior to sequencing, we determined nuclear DNA content of *C. arborea* by flow cytometry using the FACSAria II cell sorter (BD Bioscience). Nuclei suspensions were prepared using Otto buffers [14], supplemented with 50 $\mu$g/ml of RNase A solution and 50 $\mu$g/ml propidium iodide solution. Fluorescence was measured using a blue laser (488 nm), a 616/23 nm band-pass filter, and a 610 LP mirror. Based on the ratio between the mean value for the 8C peak of the internal standard *Arabidopsis thaliana* Col-0 and the mean value of the 2C sample peak, the haploid genome size of *C. arborea* was estimated to be $\sim$550 Mbp.

#### DNA isolation, 10× library preparation, and sequencing

Plant leaves were ground in liquid Nitrogen, and 51 mg powder from *C. arborea* and 56 mg from *R. gorgonias* were used for DNA extraction with the Power Plant Pro Kit (Qiagen); the Phenolic Separation Solution was used for extraction, and a vortexing step was used after RNA digestion. Tape Station results showed a peak of 20,504 bp for *R. gorgonias* and 27,474 bp for *C. arborea.*

Libraries were prepared with the Genome Protocol Kit from the Genome Reagent Kits (10x Chromium) and were quantified using the NEBNext Library Quant Kit (New England Biolabs). They had DNA concentrations of 1.78 nM (*C. arborea*) and 4.2 nM (*R. gorgonias*). Libraries were sequenced on an Illumina NextSeq 500 platform using 2 x 150 bp paired-end sequencing; this resulted in 537M (*C. arborea*) and 177M (*R. gorgonias*) reads.

#### Genome assembly

The Supernova software (10× Genomics) was used to extract fastq files and generate de novo assemblies. For the assemblies, data was subsampled to 350M reads for *C. arborea* and to 115M reads for *R. gorgonias*. Scaffolds of at least 1,000 bp were output using the Supernova pseudohap style, which represents an arbitrary mix of maternal and paternal alleles.

#### Identification and removal of contamination

Assemblies were compared to a custom database of reference genomes available from NCBI. For this analysis, only complete genomes were retrieved for bacteria, while no such restriction was used for the other divisions. This resulted in a dataset comprising 886 genomes from archaea, 293 from bacteria, 188 from invertebrates, and 94 from protozoa. As an alternative database, a local installation of Genbank’s nt (v.230) was used. The software BLAST [15, 16] was used to separately compare *C. arborea* and *R. gorgonias* scaffolds to these two databases with an E-value threshold of $10^{-15}$ and a maximum of 10 target sequences. The resulting tables were imported into MEGAN6-LR [17], and scaffolds were read in as long-reads.

#### Genome annotation using MAKER

RepeatModeler (http://www.repeatmasker.org/RepeatModeler/) was used to identify species-specific repeats for *R. gorgonias* and *C. arborea*. The resulting libraries of repeats were used for the subsequent genome annotation steps. For structural genome annotation, MAKER [18] was run iteratively: during the first round, 42,988 predicted protein sequences from *Actinidia chinensis* [19], 34,015 from *Actinidia eriantha* [20], and 42,509 UniProt [21] sequences from plants (sprot division only) were used as evidence for homology-based gene prediction. Results from this first run were used to train SNAP HMMs. These, as well as Augustus HMMs from a BUSCO run on the assembled scaffolds were used for a second round of gene predictions. Results were used to re-train SNAP and Augustus HMMs, and these were used for a third and final round of gene predictions.

#### Gene family estimation and analysis

To assign the predicted proteins of *C. arborea* and *R. gorgonias* to orthologous gene families of other Ericales’ genomes for which predicted proteins were available, the algorithm of the OMA (Orthologous MAtrix) project [22] was used. Genome- and transcriptome- based studies we included are listed in Table  1. Protein predictions from genome assemblies were directly used. For four transcriptome data sets, TransDecoder [23] was used to identify the single best open reading frame per transcript, resulting in the total numbers of predicted proteins given in Table  1. For *Diospyros lotus*, however, this resulted in 219,698 predicted proteins, which clearly is an overestimation and would have had to be filtered. Therefore, this species was excluded from our analysis. For other sequenced plant genomes and transcriptomes within the Ericales, such as *Argania spinosa* [24], *Monotropa hypopitys* [25], and *Embelia ribes* (unpublished; direct submission of contigs), no predicted proteins were available for download, and these species were therefore also excluded. As outgroup for phylogenetic analyses, we included 44,655 predicted proteins of *Daucus carota* [26].

**Table 1:** Summary statistics for gene total numbers and lengths of the full data sets used for the inference of gene families

	Min	Median	3rd Qu	Max	Total	Type	Reference
*A. chinensis*	4	353	535	5453	34,015	g	[19]
*A. eriantha*	2	268	438	5498	42,988	g	[20]
*C. arborea*	14	324	520	4973	31,129	g	This study
*Ca. sinensis*	29	325	515	5786	76,698	g	[33]
*D. carota*	29	399	601	5453	44,655	g	[26]
*P. veris*	23	366	544	4732	18,301	g	[34]
*P. vulgaris*	49	375	605	5347	28,441	g	[35]
*R. gorgonias*	21	325	509	5314	22,655	g	This study
*S. psittacina*	39	108	155	447	22,690	t	[7]
*S. purpurea*	41	111	158	831	18,748	t	[7]
*V. macrocarpon*	40	118	212	2061	34,789	t	[36]
*Di. lotus*	40	74	107	4354	219,698	t	[37]

These protein sequences were used as input for the standalone OMA pipeline [27], and a total of 63,256 gene sets for which all pairs are inferred to be orthologs (“OMA groups”) were generated. The OMA algorithm computes high-quality orthologs but tends to output more and smaller gene families than other approaches [27]. This was also observed here, with 72.5% of the OMA groups containing sequences of five or fewer species. For further analysis we selected the 4,901 groups that contained representatives of at least 7 of the 10 ingroup species and also included a *Daucus carota* sequence. For each of the 2434 OMA groups in which all genome-based species were present (see below), we computed a multiple sequence alignment using MAFFT v7.455 [28] and a Maximum Likelihood phylogeny using RAxML v8.2.12 [29] using the PROTGAMMAWAG model and the *D. carota* sequence as outgroup.

### Results and discussion

We sequenced and assembled the nuclear genomes of the carnivorous plant *R. gorgonias* and the non-carnivorous *C. arborea*. Summary and quality metrics of the final assemblies were generated using quast [30] and are shown in Table  2.

**Table 2:** Summary statistics for scaffolds and predicted genes. Metrics are listed for scaffolds of at least 1 kbp as determined using the quast software

metric	*R. gorgonias*	*C. arborea*
Total length (>= 10 kbp)	235,721,577	437,604,713
Total length (>= 25 kbp)	200,375,750	384,820,916
Total length (>= 50 kbp)	125,205,191	312,317,250
# contigs	20,623	29,265
Largest contig	191,047	616,539
Total length	284,273,507	511,026,369
GC (%)	36.60	38.50
N50	46,982	67,174
# N’s per 100 kbp	734.67	2,082.52
Total BUSCO groups searched	2121	2121
Complete BUSCOs	1787 (84.2%)	1899 (89.5%)
Complete & single-copy BUSCOs	1712 (80.7%)	1744 (82.2%)
Complete & duplicated BUSCOs	75 (3.5%)	155 (7.3%)
Fragmented BUSCOs	203 (9.6%)	135 (6.4%)
Missing BUSCOs	131 (6.2%)	87 (4.1%)

BUSCO statistics are based on 2,121 single-copy orthologs of eudicots for the predicted protein sequences of *R. gorgonias* and *C. arborea*

#### Identification and removal of contamination

No scaffolds from *C. arborea* were assigned to any of the non-plant lineages. For *R. gorgonias*, the same six scaffolds were assigned to insects with both databases, and four additional scaffolds were assigned to insects using the custom database. These ten *R. gorgonias* scaffolds ranged in size from 1.3 kbp to 45.3 kbp and were assigned to Neoptera, Holometabola, Diptera, or Schizophora using the custom database. They had a cumulative length of 92.5 kbp and were removed from the assembly for subsequent analyses. The resulting *R. gorgonias* assembly is approximately 100 Mbp larger than an estimation based on Feulgen microdensitometry [12].

#### Genome annotation using MAKER

The final set of predicted genes using MAKER consisted of 31,129 genes for *C. arborea* and 22,655 for *R. gorgonias*. The BUSCO software [31] was used to evaluate completeness of the two annotations. Of 2,121 near universal single copy orthologs of eudicots (datasets based on OrthoDB release 10), 89.5% were identified for *C. arborea* and 84.2% for *R. gorgonias*. Full BUSCO statistics are provided in Table  2.

#### Evaluation of gene families

Using as input the predicted genes from *R. gorgonias* and *C. arborea*, together with public protein sets from genome-scale data for other Ericales’ genomes and *D. carota* as an outgroup, we generated a phylogenomic data set of OMA groups that correspond to 1:1 orthologs. We evaluated the representation of each of the 10 ingroup species in the selected OMA groups. Species for which a genome assembly was available were missing from 1% (*Actinidia chinensis*) to 10% (*Primula vulgaris*) of the groups. The three transcriptome assemblies, however, showed a considerably higher level of missingness between 64% (*Vaccinium macrocarpon*) and 82% (*Sarracenia purpurea*). We observed 121 distinct patterns of species absence and presence. The most frequently observed pattern, found in 2,434 of the selected 4,901 OMA groups, contained representatives from all the genomes and none of the transcriptomes.

Although the selected species span large evolutionary distances, and lineage-specific loss or divergence is expected to occur in some gene families, the dramatic difference in absences points to a systematic bias of transcriptomes. RNA-Seq data corresponds to transcripts that are expressed only in a given tissue at a given time, and not all protein-coding genes are therefore represented in a transcriptome assembly. In addition, sequencing errors, alternative splice forms, and paralogs present serious challenges for the assembly of transcriptomes, often resulting in unrealistically large numbers of small transcripts. To reduce this number, different filtering strategies are commonly applied, such as removing lowly expressed transcripts or collapsing transcripts based on sequence identity. The resulting number of transcripts frequently is much closer to the expected number of the organism’s (expressed) genes. However, problems remain, since many challenges of transcriptome assemblies cannot be overcome using a post-assembly filtering approach: Most transcripts still are just gene fragments, and, moreover, bona fide genes are frequently filtered out [32]. This was also observed here, with much longer predicted genes from genome data than from transcriptome data (Table  1), and with most of the sets of orthologs missing in species represented by a transcriptome assembly.

**Fig. 1:**
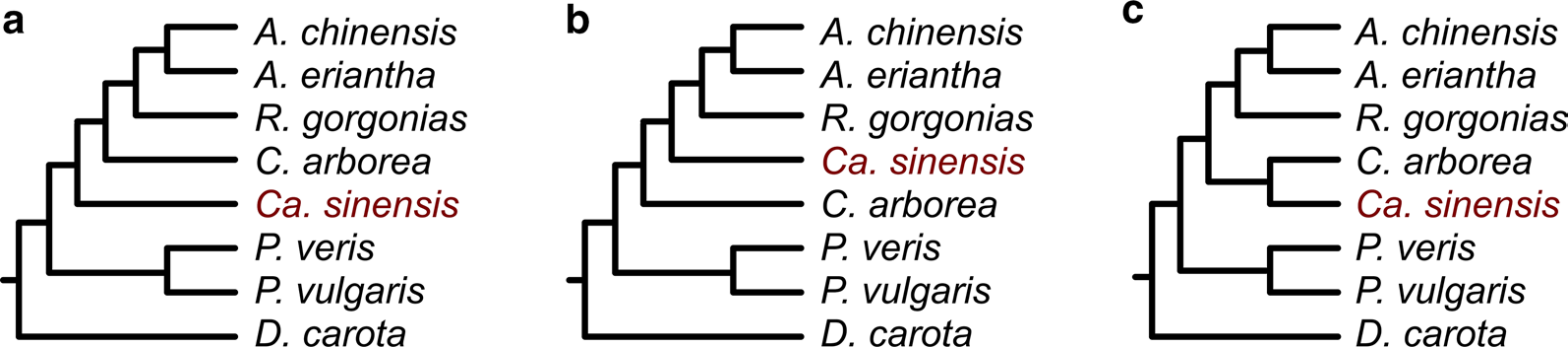
The most frequently observed ML topologies. Of 2,434 selected OMA families, 814, 218, and 176 trees resulted in the topologies shown in A, B, and C, respectively

Despite the reduced number of species in our final phylogenomic data set, we used it to infer organismal relationships within the Ericales. The 2,434 computed phylogenies revealed 118 different tree topologies, 90 of which were observed fewer than 10 times. The three most frequently observed topologies together accounted for 50% of the trees; these differ only with respect to the placement of *Camellia sinensis* and are shown in Figure  1. More genome-based data that include all lineages within the plant order Ericales are needed to confidently resolve their relationships in the future.

## Limitations

In summary, we present annotated genomes of the carnivorous plant *R. gorgonias* and the non-carnivorous relative *C. arborea*. The lengths and numbers of their predicted genes fall entirely within the range of other genome-based data and can be used to study shared and unique adaptations of plant carnivory at the molecular level. Once additional genomes, rather than transcriptomes, of other carnivorous and non-carnivorous plants within the Ericales are available, the evolution of the different carnivorous adaptations and the relationship of major lineages in this group can be resolved. Limitations of our data set are those inherent in any draft genome: due to the fragmented nature of the assembly, some genes at the end of scaffolds are likely incomplete, scaffolds corresponding to organellar genomic regions might be contained within the assembly, and repeat regions might be missing or misassembled.

## Data Availability

Raw sequence data have been deposited in the Short Read Archive under BioProject ID PRJNA630565 (https://www.ncbi.nlm.nih.gov/sra/?term=PRJNA630565), with accession numbers SAMN14840030 (*R. gorgonias*) and SAMN14840031 (*C. arborea*). Genome assemblies and predicted proteins have been made available at DataDryad under the URL https://doi.org/10.5061/dryad.573n5tb4k.

## Abbreviations

BUSCO: Benchmarking Universal Single-Copy Orthologs
DNA: Deoxyribonucleic acid
HMMs: Hidden Markov Models
IPEN: International Plant Exchange Network
kbp: Kilo base pair
M: Million
Mbp: Mega base pair
NCBI: National Center for Biotechnology Information
nm: Nano meter
RNA: Ribonucleic acid
OMA: Ortholog Matrix Project
